# A Novel 6G Conversational Orchestration Framework for Enhancing Performance and Resource Utilization in Autonomous Vehicle Networks

**DOI:** 10.3390/s23177366

**Published:** 2023-08-23

**Authors:** Sonia Shahzadi, Nauman Riaz Chaudhry, Muddesar Iqbal

**Affiliations:** 1 Department of Computer Science, University of Gujrat, Gujrat 50700, Pakistan; 18036119-002@uog.edu.pk; 2 Renewable Energy Laboratory, Communications and Networks Engineering Department, College of Engineering, Prince Sultan University, Riyadh 11586, Saudi Arabia

**Keywords:** 6G, MEC, XaaS, QoS, LXC, NLU, AI

## Abstract

A vision of 6G aims to automate versatile services by eliminating the complexity of human effort for Industry 5.0 applications. This results in an intelligent environment with cognitive and collaborative capabilities of AI conversational orchestration that enable a variety of applications across smart Autonomous Vehicle (AV) networks. In this article, an innovative framework for AI conversational orchestration is proposed by enabling on-the-fly virtual infrastructure service orchestration for Anything-as-a-Service (XaaS) to automate a network service paradigm. The proposed framework will potentially contribute to the growth of 6G conversational orchestration by enabling on-the-fly automation of cloud and network services. The orchestration aspect of the 6G vision is not limited to cognitive collaborative communications, but also extends to context-aware personalized infrastructure for 6G automation. The experimental results of the implemented proof-of-concept framework are presented. These experiments not only affirm the technical capabilities of this framework, but also push into several Industry 5.0 applications.

## 1. Introduction

6G is expected to automate interdisciplinary domains, including industrial production, health, education, transport, business, and infrastructure. The concept of industrial automation has attracted the attention of 6G communication, given the continuous demand from each industry sector to fully automate the process of manufacturing [1]. The automation of the process, manufacturing goods and services with collaboration between cobots and humans, is presented to support the Industry 5.0 concept [2]. Industry 5.0 will enable the real-time interaction between humans and machines with collaborative cognitive computing [3]. Artificial Intelligence (AI) is expected to be the preferred area for 6G automation by expanding industrial Internet of Things (IoT) applications with sensing capabilities and learning requirements that utilize distributed federated learning. The actual required goal of 6G automation is the elimination of human influence from production, and the full automation of systems and services to reduce the probability of human error [4]. Ensuring strict latency requirements and reliable communication remains crucial for live response/feedback when massively connected devices combine to achieve a shared goal. Therefore, 6G technology will be used to collect fast and reliable responses from each module, to fulfill the required objective [1]. With the rapid commercialization of network technologies, 6G will convert business scenarios and society activity to connected intelligence and drive 6G-inspired business transformation.

With the rapid development of mobile technologies across multiple generations of networks, a sustainable 6G kiosk is in high demand to deliver cutting-edge shared experiences as well as heterogeneous services [5]. Standardization and regulation work will affect the future of 6G technology, protecting individual identity and privacy, which remains a long-term challenge across multiple nations with different perspectives [6]. To ensure the trustworthiness of the 6G vision, and to maintain the trust of the end user, multidisciplinary research challenges, including techno-politics, techno-economics, and techno-ethics, are necessary to deal with sustainability and social legislation [7]. This will draw a broad roadmap of the physical, biological, and digital sensing world. Regulation policies are used to describe the behavior of each node, and collective evidence behavior from ubiquitous devices may scale the applications in multiple domains to produce productive outcomes [6]. The rapid commercialization of 5G that started in around 2020 across the world is not only used to enhance communication, but also to contribute to the design of customized intelligent services. 6G is expected to continue this transformation from the connected-society era to the connectivity of everything with connected intelligence. By fusing physical, digital, and biological worlds, 6G will usher in a significant advancement that will revolutionize the digital age [5]. This will contribute to achieving a multi-sensory experience for the Industry 5.0 revolution, which will ensure innovations and applications for societal well-being [8]. By considering the human aspects of life, 6G communication is expected to go beyond traditional communications by enabling the true intelligence of everything, including individuals, businesses, and homes, forming the horizons of revolutions. It will sense information from the environment and bring a holistic view of distributed modules. AI and sensing in 6G are considered to be a new era for connected people aimed at connected intelligence. Haptic feedback, designed to adapt human-centric applications and holographic displays for Extended Reality (XR), will potentially increase network traffic demand among massively connected intelligent devices [8]. Federated learning at the edge may accomplish critical performance requirements with deeply converged ICT systems for diverse computing, intelligence, and connectivity at the edge. This enlarges the existing centralized cloud AI towards edge AI. Targeting the largest number of emerging services with 6G scenarios will increase additional overheads on networks and devices. Industrial verticals will find it arduous to achieve their desired goals and fulfill their production and application requirements. Therefore, telecommunication operators are targeting the progress of AI, cloud, and micro-services. Accordingly, industry and academia are making huge efforts regarding these concept adaptations to achieve their desired Key Performance Indicators (KPIs). In our previous work, we presented Collaborative Cognitive Communication (3C) systems that can enable human-centric and adaptive services for Industry 5.0 applications over 6G networks [9]. In this article, we present an on-the-fly virtual orchestration framework to leverage the concepts of AI, cloud, and micro-services that can contribute to 6G orchestration and automation. The contributions of this article are:

1. A novel proposed on-the-fly framework that is aligned with conversational AI assistance and automation;
2. A conversational orchestrator to convert non-technical requirements into technical functional requirements using the Cloud Native Environment (CNE), Natural Language Processing (NLP), and micro-services;
3. A performance evolution of the suggested framework to make it reliable and scalable.

## 2. Related Work

Several academic researchers and industrial players have been working on developing virtual infrastructure management to control cloud application requirements. A new routing protocol for Connected and Autonomous Vehicles (CAV) over 6G networks has been presented in this article [10]. This protocol can deliver excellent data coverage and service quality in real-time scenarios. The protocol uses a risk-aware security mechanism to assure session-oriented communication and prevent uncertainties in the autonomous system, as well as a simulated annealing optimization technique to increase energy economy and dependability among IoT-based vehicles [10]. A framework and architecture for a swarm of Unmanned Aerial Vehicles (UAVs) for network management in 6G has been suggested in this article [11]. It analyzes the opportunities and problems of combining AI, IoT and blockchain technologies with UAV networks [11]. In order to provide MEC services for large-scale User Equipment (UE) in 5G/6G networks, this study presented a new system that combines multiple Intelligent Reflecting Surfaces (IRSs) with multiple UAVs [12].

Highly automated and orchestration architecture is one of the prominent KPIs in emerging technologies including Fifth Generation (5G) service deployment and the Mobile Edge Computing (MEC) paradigm [13,14]. XaaS is becoming a popular term, but creating some misconceptions; XaaS refers to the universe of the entire cloud to deliver services, while another model defined XaaS as referring only to those services that already have been deployed on the cloud or will be transferred to the cloud [15]. This confusion is clear from the state of the art of different XaaS models, and general cloud service models IaaS, PaaS and SaaS have contributed significantly to the formation of XaaS model development. As cloud computing offers many virtualized resources and services, self-managing resources for different cloud applications are required to optimize the QoS specifically under the XaaS paradigm. To deal with this problematic behavior, different cloud resource orchestration and automation frameworks have been proposed to help selection and auto-deployment of resources in [13,16,17,18,19,20]. To study the technical and analytical dimensions, we have investigated existing frameworks from the latest research.

A component-oriented approach called the Method for AutomateD prOvisioning of cloud-based component-oriented busiNess Applications (MADONA) has been suggested to allow for the automatic provisioning of cloud business applications [21]. It fulfills the orchestration phase of cloud applications using Juju, and provides technical comparisons with Bitnami and Juju [21]. In [22], the Open-source API and Platform for Multiple Clouds (mOSAIC) middleware solution has been proposed to support the cloud portability of applications among federated cloud environments. However, it fails to ensure security issues among multiple clouds. It provides two basic selection scenarios: one for developing an application from scratch, and the other for migrating an existing application. However, automated provisioning of applications is a challenging task in mOSAIC. In [23], the Smart Applications on Virtual Infrastructure (SAVI) testbed has been proposed to manage virtual Information and Communication Technology (ICT) resources and address the future applications market design. It is built on the Virtualized Application Networking Infrastructure (VANI) testbed used to deploy networking infrastructure and distributed applications on the virtualized resources. The SAVI testbed consists of five major components, i.e., smart edge nodes, core nodes, access nodes, SAVI TB control center and SAVI network. All these nodes (i.e., core, smart edge and access nodes) are used to create and host applications, and the SAVI network provides interconnected networking of all these components. The SAVI TB control center functionalities are authentication and authorization. A toolkit has been proposed for infrastructure automation, which consists of a network infrastructure manager, deployment manager, platform setup manager and service manager [24].

Primarily frameworks mentioned above do not consider QoS except for MODANA, but MODANA QoS is based on weight instead of data integrity and high availability. It does not provide any precise value for QoS parameters. moSAIC is the only another framework that provides on-the-fly functionality, but it does not have the containerization to improve the QoS. Table 1 and Table 2 show a detailed analytical and technical comparison of the existing frameworks mentioned above. To overcome the existing limitations and improve the existing solution, we have proposed a solution that does not only overcome these limitations, but also provides a XaaS framework via an on-the-fly mechanism and containerization technology for QoS improvements.

## 3. Proposed Framework

**Figure 1:**
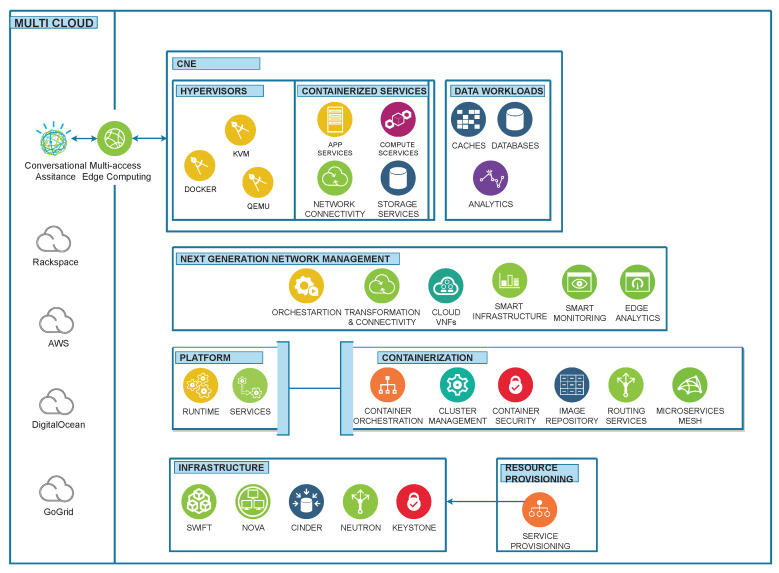
Architecture of proposed framework.

**Figure 2:**
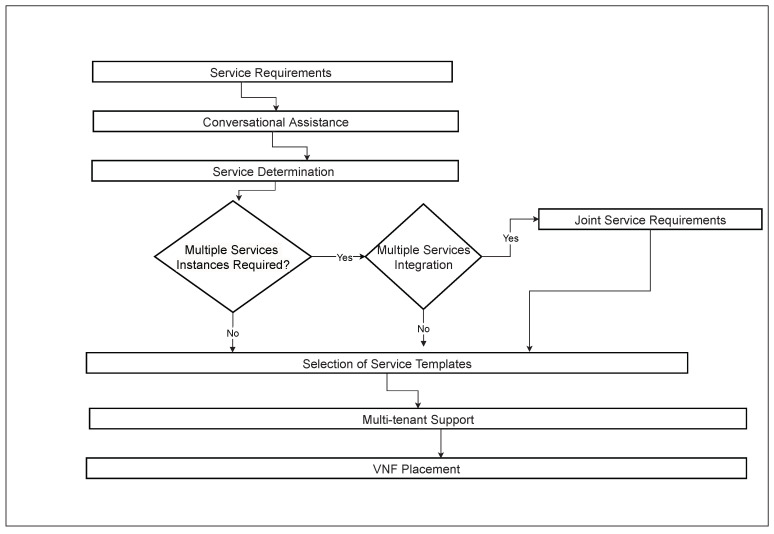
Service flow of proposed framework.

The 6G vision is to fully connect the world by exploring novel architectures and decomposition into micro-services, functions and orchestration aspects. The execution of this framework is based on a real-time testbed implemented to achieve the dream of XaaS. The requirements from end users are passed to the cloud via a multi-cloud orchestration API designed to support centralized and distributed resource management. This framework initiates the deployment of Virtual Network Functions (VNFs) based on in-demand requests of users and on-the-fly VNF placement mechanisms executed to fulfill the requested services. KVM, QEMU and docker are supported as hypervisors to execute the service on the cloud. More specifically, Linux Containers (LXCs) are additionally embedded as hypervisors to reduce the response time of services. To improve the QoS, lightweight containerization technology is used and a docker is implemented as a hypervisor of the instance. The additional computing resources, such as CPUs, RAM and networks, are assigned against specific instances of the cloud. To maintain the minimum level of QoE, this solution acts as a primary controller of resources to respond to the live request and triggers specific actions against user requests. In this work, we have developed a framework for XaaS to support automated resource provisioning on-the-fly with containerization technology and on-demand service access with improving QoS. This proposed framework has been designed to automate resource provisioning with containerization technology, which will provide on-demand services by improving QoS under the XaaS paradigm. A high-level view of proposed architecture towards on-the-fly 6G orchestration has been presented in Figure 1, and the service flow of this framework has been presented in Figure 2. In Figure 2, initially, it required high-level requirements from the end user. After taking service requirements, the execution of the framework will start to determine the service type. This determination will finalize the module for single or multiple instances, and proceed according to the requirements. If multiple service instances are required to fulfill the user demand, it will join the multiple service requirements to create the desired service against each user. These joint service requirements are executed inside the multi-tenant support. After that, VNF placement will be assigned to the specific required service.

## 4. Implementation

To fulfill the requirements of conversational orchestration in 6G, a prototype has been developed using CNE and AI technologies. We have used an Ubuntu server as the host OS and OpenStack as a cloud provider. Multi-cloud SDKs have also been used, to make it compatible with other cloud providers. Rasa NLU and Rasa Core have been used to achieve the conversational AI requirements, while customized intents can be designed using the GUI interface and the AI models trained using the NLU unit. Using an AI assistant, a user may fulfill their criteria without utilizing any technical knowledge with a conversational chatbot. These requirements are forwarded to a cloud orchestration API that is also compatible with centralized and distributed resource management. The orchestrator initiated the on-the-fly mechanism and placed the VNF mechanism against specific user requests. The designed framework is compatible with KVM/QEMU and LXC hypervisor, and can appoint additional resources (i.e., vCPUs, RAM, networks) to continue the minimum QoE level. We have used the cloud orchestrator as a central controller using LibCloud SDK, and services are deployed via VMs/Containers through a conversational interface. To improve the QoE, migration of services from high-load regions/nodes to underutilized regions with traffic balancing is possible. Failure of any single node is transparently dealt with on-the-fly without the users knowing.

For cloud development, we have used the OpenStack platform, which is flexible and extended, and provides LXC compatibility to support our solution. For lightweight technology implantation, we have used a docker, as it has become the most popular containerization technology. The cloud infrastructure manager will manage all the operations of cloud services. In contrast, a cloud service broker will deliver the communication services among multiple components of clouds, and a cloud orchestrator will provide the resource provisioning services. The tools and technologies required to implement this testbed have been represented in Table 3 with descriptions.

## 5. Performance Evaluation

We have performed XaaS Experiments with different KPIs, including service authentication time, network time, service deployment time, VNF assignment time and service migration time. The size of the services deployed in the cloud can have an impact on a number of things, including:

1. Performance: The image size may influence the VMs’ boot times, memory requirements, and disk space. Larger images may require more disk space, memory, and longer boot times than smaller images. The usefulness and efficiency of the VMs can be enhanced by the additional features and apps that may be presented in larger images;
2. Cost/price: Since some cloud providers base their prices on the amount of storage and bandwidth an image uses, their size can impact how much a cloud service will cost. Due to their higher storage and bandwidth requirements, larger images could have higher expenses than smaller images.

Comparing various image sizes for cloud service deployments would entail weighing their benefits and drawbacks, contrasting them with other techniques and tools, and determining how well-suited they are to various scenarios and configurations. The description of experimental services to validate the framework has been presented in Table 4. The terms that have been used in experimental figures are represented below:

1. Total duration: Indicates how long it takes to complete the scenario as a whole and each atomic action;
2. Min: The minimal value of gained time for iterations of the scenario/atomic operation;
3. Max: The amount of time spent on scenario/atomic action iterations is the greatest;
4. Average: The median is the average time spent on scenario/atomic action iterations;
5. 90%ile: 90% of scenario/atomic action iterations acquired time less than this value, making it the lowest duration value;
6. 95%ile: The lowest duration number is 95%ile, which indicates that 95% of scenario/atomic action iterations took longer than this amount of time;
7. Success: A 100% success rating will be displayed if a particular scenario runs successfully throughout all iterations. Failure is not a 100% guarantee of success if some iterations are unsuccessful;
8. Count: a display of the total number of iterations.

### 5.1. Service Authentication Experiments

**Figure 3:**
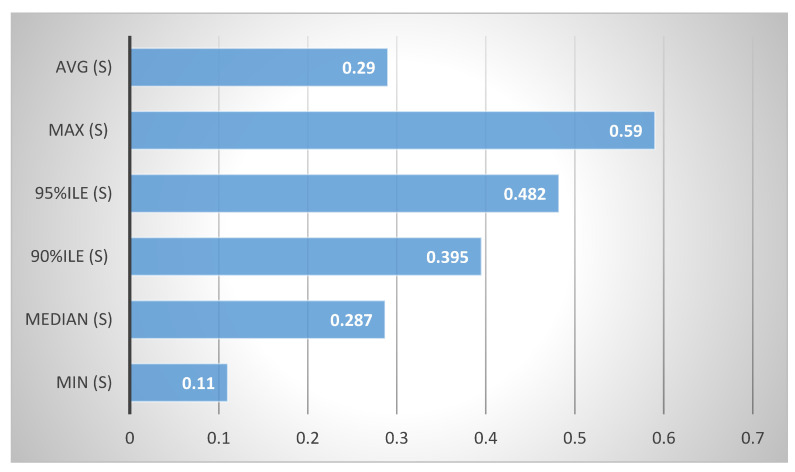
Authentication experiments.

The authentication mechanism offers a central user directory linked to the cloud services that users can use, making managing and administering identity data easier. It supports a variety of backends and authentication methods, including SQL, LDAP, OAuth, OpenID Connect and SAML, giving users and services flexibility and compatibility. It offers a service catalog that compiles the endpoints of various cloud services, facilitating service integration and discovery. For various resources and services, it offers dynamic policies and role-based access control, improving the cloud environment’s security and governance. The support for federated identification and single sign-on for users across numerous applications and cloud services enhances the user experience and convenience. Service authentication time has been measured against 50 iterations, with five concurrency in authentication experiments. The experiment has been completed in an average of 0.29 s, 0.11 s with minimum time and 0.59 s with maximum time against 50 iterations, as presented in Figure 3.

### 5.2. Network Experiments

**Figure 4:**
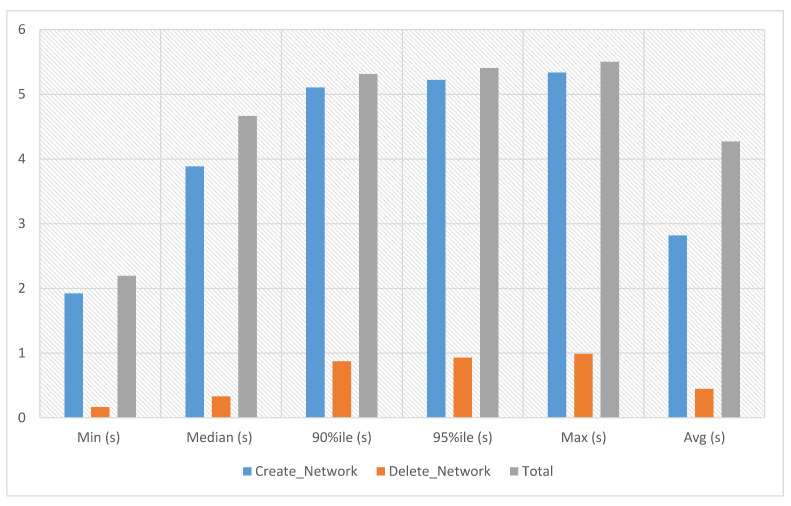
Network experiments.

The network module support enables the users to construct and manage network objects that other cloud services can use, including networks, subnets and ports. Project and provider networks are two available network types that users can share as part of the network construction procedure. When networks are no longer required, users can delete them, freeing up space and reducing costs. In network experiments, network creation and deletion time has been calculated against ten iterations. The experiment time against ten iterations is an average of 4.27 s, a minimum of 2.194 s and a maximum of 5.502 s, as presented in Figure 4.

### 5.3. Service-1 Deployment Experiments

**Figure 5:**
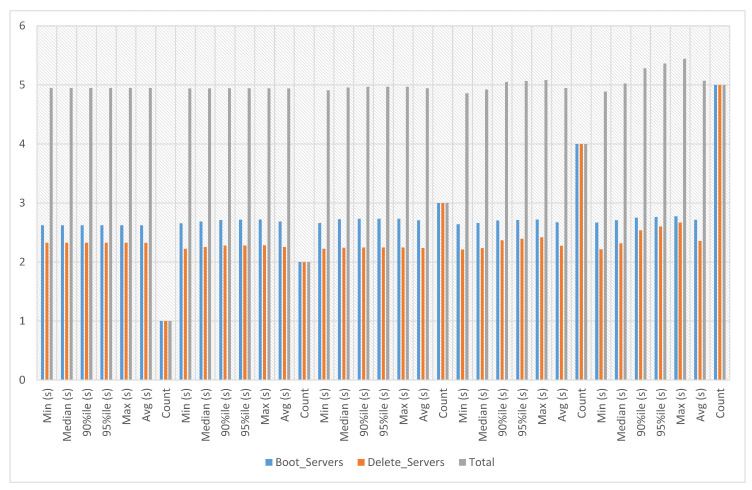
Service-1 Experiments.

In the service-1 experiment, a service image size of 371.8 MB is used with the m1.large flavor to launch the service instance. The average, minimum and maximum experiment times for a single iteration are 4.951 s. The experiment time against two iterations is an average of 4.943 s, a minimum of 4.942 s and a maximum of 4.944 s. The experiment time against three iterations is an average of 4.945 s, a minimum of 4.91 s and a maximum of 4.968 s. The experiment time against four iterations is an average of 4.948 s, a minimum of 4.861 s and a maximum of 5.085 s. The experiment time against five iterations is an average of 5.07 s, a minimum of 4.888 s and a maximum of 5.443 s. The service-1 experiment time has been presented in Figure 5, where the x-axis denotes the number of iterations and the y-axis the total time required to finish each iteration.

#### VNF Assignment to Service-1

**Figure 6:**
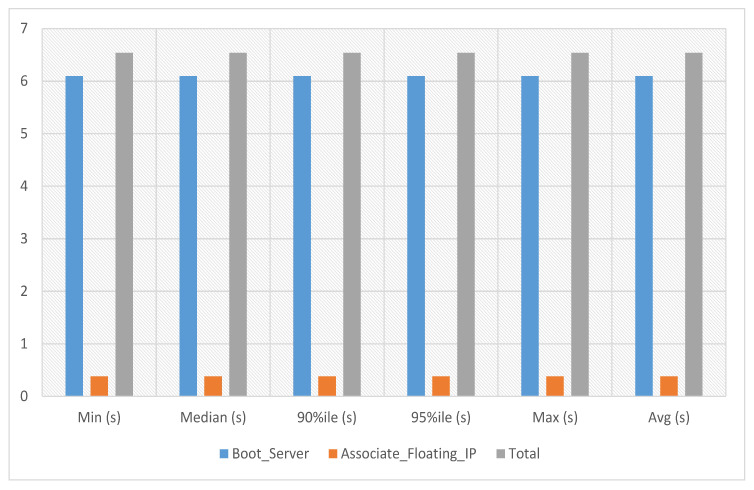
VNF assignment to service-1.

VNFs are network functions in a cloud environment that run on software rather than the more conventional hardware in a cloud environment. VNFs can provide several advantages for cloud services, including scalability, agility, resource optimization, cost savings, and improved security and management. In the VNF assignment to service 1 experiment, the server’s boot time with floating IP association against this service-1 has been calculated. The average, minimum and maximum time against a single iteration is 6.541 s as presented in Figure 6 where the x-axis indicates the number of iterations and the y-axis the total time required to complete a given iteration.

### 5.4. Service-2 Deployment Experiments

**Figure 7:**
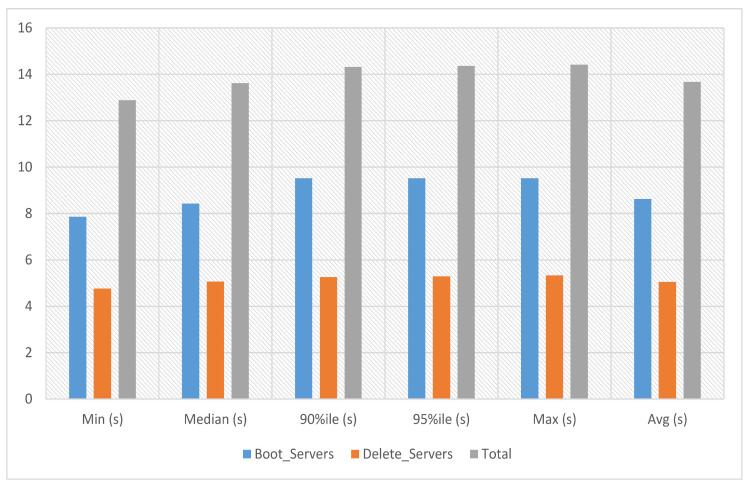
Service-2 experiments.

In service-2 experiments, a service image size of 12.6 MB is used with the m1.tiny flavor to launch the service instance. This service’s boot and delete time on multiple servers has been calculated with count 1. The experiment time against ten iterations is an average of 13.674 s, a minimum of 12.885 s and a maximum of 14.409 s, as presented in Figure 7.

#### 5.4.1. VNF Assignment to Service-2

**Figure 8:**
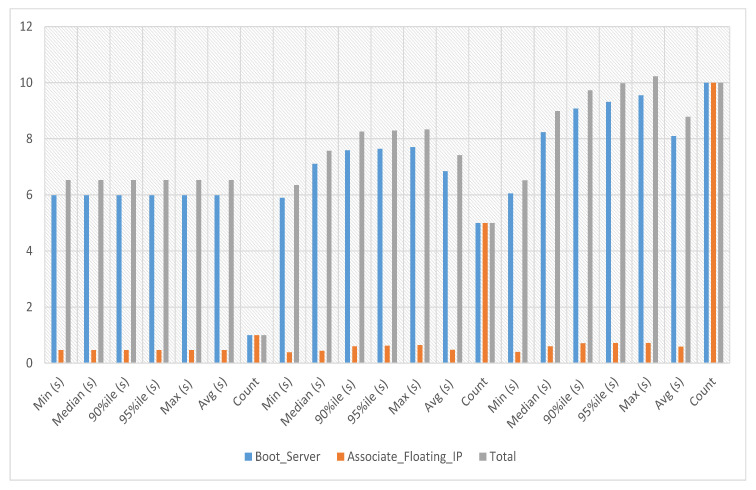
VNF assignment to service-2.

In VNF assignment to service-2 experiments, the server’s boot time with floating IP association against service-2 has been calculated against iterations 1, 5 and 10, respectively. The average, minimum and maximum time against a single iteration is 6.533 s. The experiment time against five iterations is an average of 7.419 s, a minimum of 6.354 s and a maximum of 8.334 s. The experiment time against ten iterations is an average of 8.78 s, a minimum of 6.524 s and a maximum of 10.229 s as presented in Figure 8, where the y-axis represents the total time needed to complete a specific iteration and the x-axis represents the number of iterations.

#### 5.4.2. Service-2 Migration Experiments

**Figure 9:**
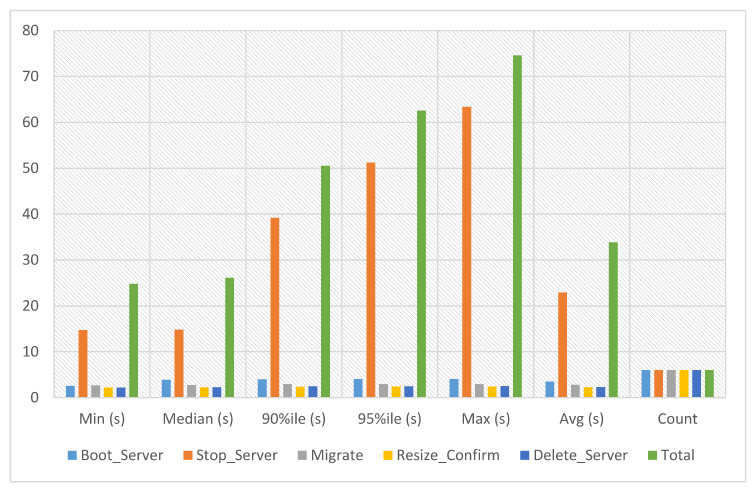
Service-2 migration experiments.

Using the migration service, instances can be transferred from one computer server to another without termination. Users can maximize resource utilization, avoid service interruptions, and improve performance and availability by moving instances. In service-2 migration experiments, the migration time of service-2 has been calculated against six iterations. The experiment time against six iterations is an average of 33.853 s, a minimum of 24.792 s and a maximum of 74.607 s, as presented in Figure 9.

### 5.5. Service-3 Deployment Experiments

**Figure 10:**
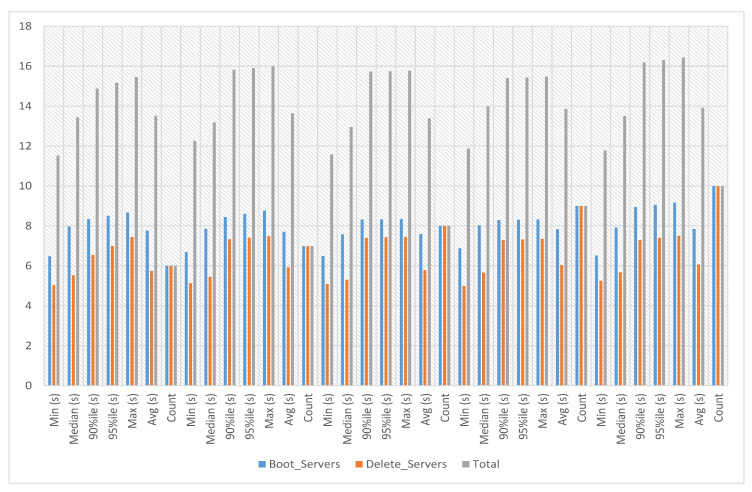
Service-3 experiments.

In service-3 experiments, a service image size of 23.5 KB is used with the m1.tiny flavor to launch the service instance. This service’s boot and delete time on multiple servers has been calculated against iterations six to ten. The experiment time against six iterations with count 5 is an average of 13.52 s, a minimum of 11.529 s and a maximum of 15.461 s. The experiment time against seven iterations with count 5 is an average of 13.639 s, a minimum of 12.272 s and a maximum of 15.992 s. The experiment time against eight iterations with count 5 is an average of 13.393 s, a minimum of 11.585 s and a maximum of 15.77 s. The experiment time against nine iterations with count 5 is an average of 13.878 s, a minimum of 11.875 s and a maximum of 15.476 s. The experiment time against ten iterations with count 5 is an average of 13.923 s, a minimum of 11.777 s and a maximum of 16.435 s, as presented in Figure 10.

#### 5.5.1. VNF Assignment to Service-3

**Figure 11:**
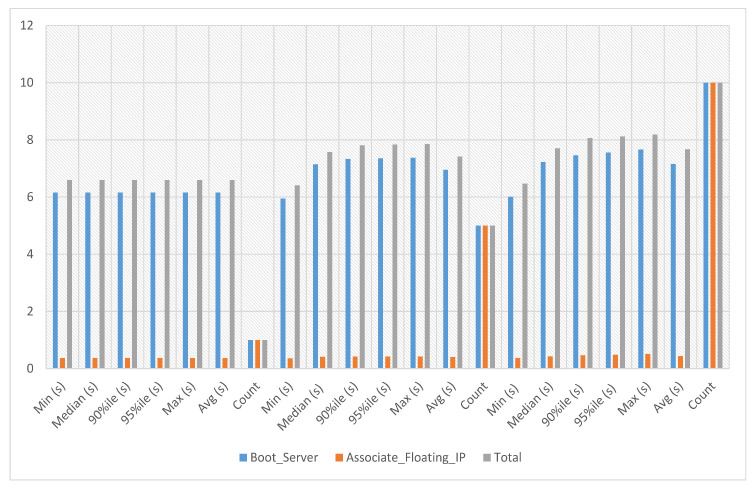
VNF assignment to service-3.

In the VNF assignment to service-3 experiments, the server’s boot time with floating IP association against this service-3 has been calculated against iterations 1, 5, and 10, respectively. The average, minimum and maximum time against a single iteration is 6.595 s. The experiment time against five iterations is an average of 7.422 s, a minimum of 6.414 s and a maximum of 7.855 s. The experiment time against ten iterations is an average of 7.67 s, a minimum of 6.471 s and a maximum of 8.188 s, as presented in Figure 11 where the y-axis shows the total time required to finish a particular iteration, while the x-axis shows the number of iterations.

#### 5.5.2. Service-3 Migration Experiments

**Figure 12:**
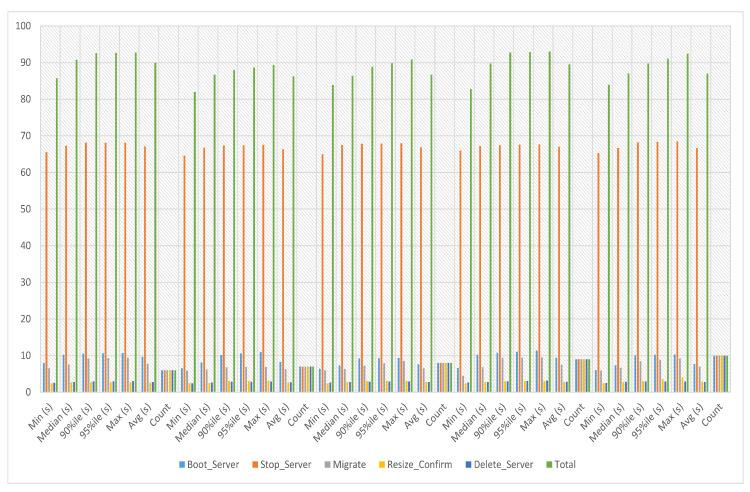
Service-3 migration experiments.

Instances can be moved from one computer host to another using the migration service without being shut down. Users can prevent service interruptions, maximize resource utilization, and boost performance and availability by transferring instances. In service-3 migration experiments, the migration time of service-3 has been calculated against iterations six to ten. The experiment time against six iterations is an average of 89.965 s, a minimum of 85.759 s and a maximum of 92.749 s. The experiment time against seven iterations is an average of 86.272 s, a minimum of 81.996 s and a maximum of 89.356 s. The experiment time against eight iterations is an average of 86.676 s, a minimum of 83.881 s and a maximum of 90.863 s. The experiment time against nine iterations is an average of 89.522 s, a minimum of 82.759 s and a maximum of 93.039 s. The experiment time against ten iterations is an average of 87.017 s, a minimum of 83.946 s and a maximum of 92.421 s, as presented in Figure 12.

### 5.6. Service-4 Deployment Experiments

**Figure 13:**
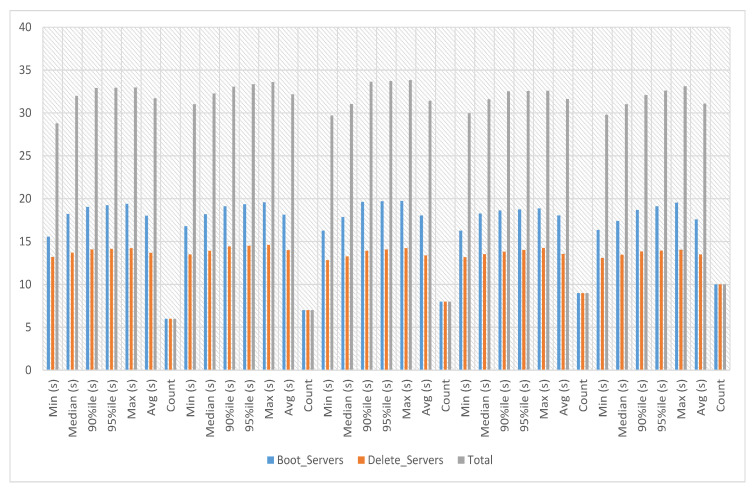
Service-4 experiments.

In service-4 experiments, a service image size of 469.3 MB is used with the m1.tiny flavor to launch the service instance. This service’s boot and delete time on multiple servers has been calculated against iterations six to ten. The experiment time against six iterations with count 5 is an average of 31.726 s, a minimum of 28.791 s and a maximum of 32.965 s. The experiment time against seven iterations with count 5 is an average of 32.163 s, a minimum of 31.026 s and a maximum of 33.612 s. The experiment time against eight iterations with count 5 is an average of 31.426 s, a minimum of 29.694 s and a maximum of 33.831 s. The experiment time against nine iterations with count 5 is an average of 31.612 s, a minimum of 29.975 s and a maximum of 32.597 s. The experiment time against ten iterations with count 5 is an average of 31.083 s, a minimum of 29.802 s and a maximum of 33.118 s, as presented in Figure 13.

#### VNF Assignment to Service-4

**Figure 14:**
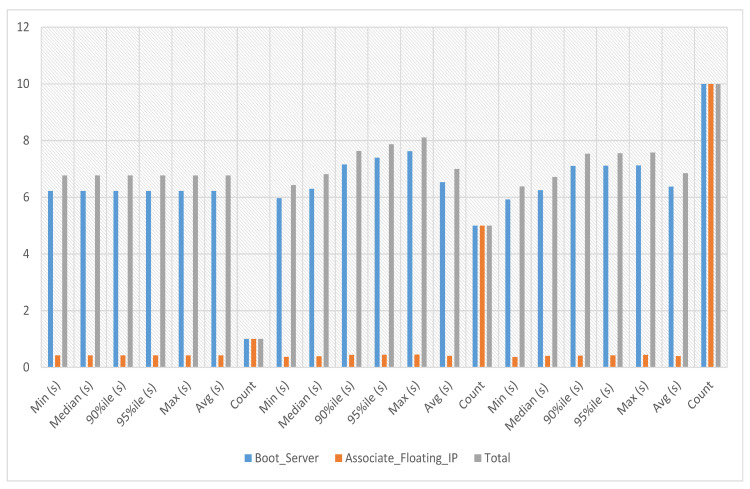
VNF Assignment to service-4.

In the VNF assignment to service-4 experiments, the server’s boot time with floating IP association against service-4 has been calculated against iterations 1, 5 and 10, respectively. The average, minimum and maximum time against a single iteration is 6.77 s. The experiment time against five iterations is an average of 7.005 s, a minimum of 6.436 s and a maximum of 8.11 s. The experiment time against ten iterations is an average of 6.847 s, a minimum of 6.387 s and a maximum of 7.579 s, as in Figure 14, where the y-axis displays the total time required to finish a particular iteration and the x-axis shows the number of iterations.

## 6. Conclusions

In this article, we proposed on-the-fly virtual infrastructure service orchestration for XaaS to automate cloud services provisioned to react on-the-fly with the required QoS in AV networks. The proposed framework takes on-the-fly feedback from the system and places VNFs for XaaS. The resulting outcomes demonstrate the technological prowess and performance of the proposed solution. It covers various aspects under the XaaS paradigm, including user/service authentication, network construction and deletion, and IaaS, PaaS, and SaaS deployment times. It demonstrates the better reliability and scalability that the suggested system can achieve. The outcomes further demonstrate that the proposed system can adapt to various scenarios and user preferences by utilizing the suggested architecture.

## Figures and Tables

**Table 1:** Analytical comparison of cloud resource orchestration and automation frameworks.

Reference	Year	Solution	Approach	Description	Benefits	Challenges
[25]	2005	Puppet	Declarative approach	Provides automated infrastructure and continues delivery	Reduce the threat of external attacks	Limited flexibility
[26]	2009	Chef	Declarative and imperative approach	Configuration management tool	High availability	Limited flexibility
[27]	2009	JCLoud	Cloud APIs and libraries	To support multi-cloud environment and portability	Run time portability	No decentralization and network infrastructure
[28]	2009	LibCLoud	Cloud APIs and libraries	To support multi-cloud environment and portability	Application portability	No decentralization and network infrastructure
[29]	2010	Juju	Orchestration based tool	Components deployment using charms	Easy and quick deployment of cloud services	Limited flexibility
[30]	2012	Ansible	Declarative and imperative approach	Configuration management tool	Easy with multi-playbook workflow	Limited flexibility
[22]	2013	mOSAIC	Cloud APIs and libraries	Multi-cloud resource management to support portability of applications	Elasticity and auto scaling	Lack of network infrastructure, security and automated management
[23]	2013	SAVI	Component-oriented approach	Built on the Virtualized Application Networking Infrastructure (VANI)	Flexible and versatile infrastructure for future applications	Requires highly technical skills
[31]	2014	Terraform	Declarative approach	Provides infrastructure as a code	Provides support for different infrastructure providers	Difficult to manage states of resources
[32]	2015	CometCloud	Layers approach	Autonomic framework to support end-to-end workflow	Heterogeneous and flexible	Lack of network infrastructure and auto scaling
[33]	2017	MADONA	Component-oriented approach	Automatic provisioning of cloud applications	Reduces technical knowledge	High provisioning time
[24]	2020	Virtual Infrastructure Orchestration	Scripts-based approach	Provides infrastructure automation of complex procedures	Reduces deployment time and minimize manual efforts	Limited flexibility

**Table 2:** Technical comparison of cloud resource orchestration and automation frameworks.

References	FC	PC	ID	NI	PS	AS	QoS	DN	OS	OF	Language
[21]	Y	Y	Y	N	N	−	Y	N	N	Y	Python
[22]	Y	Y	Y	N	N	Y	N	N	N	Y	Java
[32]	Y	Y	Y	N	N	N	N	Y	N	N	Java
[23]	Y	N	N	Y	Y	Y	N	N	N	N	Java and Python
[24]	Y	Y	Y	Y	N	Y	N	N	Y	N	Python
[31]	Y	Y	Y	Y	−	Y	N	N	Y	N	Go
[25]	Y	Y	−	N	Y	−	N	N	Y	N	Ruby
[26]	Y	Y	N	N	Y	−	N	N	Y	N	Ruby
[27]	Y	Y	N	N	Y	N	N	N	Y	N	Java
[28]	Y	Y	N	N	Y	N	N	N	Y	N	Python
[29]	Y	Y	−	N	Y	−	N	N	Y	N	Python
[30]	Y	Y	N	N	N	−	N	N	Y	N	Python

Note: FC = Federated Cloud; PC = Public Cloud; ID = Infrastructure Description; NI = Networked Infrastructure; PS = Provision from scratch; AS = Auto Scaling; DN = Decentralization; OS = Open Source; OF = On-the-Fly; Y = Yes; N = No.

**Table 3:** Tools and technologies.

S.No	Item	Description
1	Host OS	Linux Server
2	Cloud Platform	OpenStack
3	Linux Container	Docker
4	Orchestration Deployment	Multi-Cloud SDK
5	Conversational Platform	Rasa NLU/Rasa Core
6	Programming Language	Python

**Table 4:** XaaS Scenarios for proposed framework.

Services	Size	Flavor	Hypervisor	Network
Service 1	371.8 MB	m1.large	QEMU	Private
Service 2	12.6 MB	m1.tiny	QEMU	Private
Service 3	23.5 KB	m1.tiny	Docker (LXC)	Private
Service 4	469.3 MB	m1.tiny	Docker (LXC)	Private

## Data Availability

Not applicable.
